# Not guilty by association: myelodysplasia-related mutations in AML

**DOI:** 10.1038/s41375-026-02936-z

**Published:** 2026-04-22

**Authors:** Sangeetha Venugopal, Mikkael A. Sekeres

**Affiliations:** Division of Hematology, Sylvester Comprehensive Cancer Center, University of Miami, Miami, FL USA

**Keywords:** Risk factors, Haematological cancer

In law, the punishment should fit the crime, and it is the burden of the prosecution to establish *mens rea* [1]—the guilty mind—that a defendant had the necessary criminal intent to act purposely, knowingly, recklessly, and negligently.

The same can be said of precursor myeloid mutations that can lead to a diagnosis of acute myeloid leukemia (AML). But just because you run with a gang of thugs, does that also make you a thug?

It has long been recognized that discrete prognostic factors within the broad category of AML can render a life-threatening diagnosis either better, as with acute promyelocytic leukemia or AML associated with core binding factor abnormalities, or far worse [2]. For example, we have known for decades that, for people with AML, it is better to be younger than older, to have fewer comorbidities and a better performance status than to have multiple suffering organs and to be moribund, and to have a normal white blood cell count rather than one that sets new standards for “WOW” factor.

Importantly, it is also better to have a truly de novo AML, as is more common in young people with fewer co-morbidities and a better performance status, than to have an AML that has arisen from an already ailing bone marrow, as occurs more frequently in older adults with myelodysplastic syndromes/neoplasms (MDS). The European LeukemiaNet (ELN) 2022 guidelines identified myelodysplasia-related (MR)- gene variants like *ASXL1, BCOR, EZH2, RUNX1, SF3B1, SRSF2, STAG2, U2AF1*, and/or *ZRSR2* as being associated with adverse risk, with the caveat that they may not retain their poor prognostic significance if they co-occur with favorable-risk genetic variants [3]. But was this sweeping declaration justified when an individual variant frequently seen in MDS may not portend doom and may just have been guilty by association?

In this issue of *LEUKEMIA* Bill and colleagues [4] analyze data from almost 5000 subjects with AML to determine the prognostic significance of MR-variants, in essence asking the question, “are each of them really as bad as we think they are?” Subjects received intensive treatment regimens (acknowledging, in a venetoclax-based therapy era, that chemotherapy intensity is in the eye of the beholder) across five cooperative study groups, enrolled over two decades.

The authors reported that approximately one-third (34.1%) of the cohort harbored at least one MR-mutation. In line with the ELN 2022 recommendations, compared to those without MR-mutations, patients with MR-mutations had significantly worse outcomes with respect to complete remission rates (65.7% vs 77.7%; *p* < 0.001), event-free (HR 1.45; *p* < 0.001), relapse-free (RFS; HR = 1.33; *p* < 0.001), and survival (HR = 1.45; *p* < 0.001). However, those with MR-mutated AML had significantly better RFS and survival compared with those with other, non-MR adverse risk variants. As anticipated, when they co-occurred with favorable-risk features, MR-mutations did not retain their adverse prognostic significance. Among those who underwent allogeneic hematopoietic cell transplantation (HCT), those with MR-mutations had shorter overall survival compared to those undergoing transplantation without mutations.

When individual MR-gene variants were compared with ELN 2022 intermediate- and adverse-risk AML, distinct gene-specific prognostic patterns became evident. Some variants were particularly bad, like *ASXL1, RUNX1, SF3B1* and *U2AF1,* resulting in adverse risk-like outcomes. Others were merely guilt by association: *BCOR, SRSF2, and STAG2* variants weren’t great, but not awful either, with outcomes like intermediate-risk AML. Perhaps they acted recklessly and negligently but not purposely or knowingly.

The implications of this research could be to intensify post-remission regimens using approaches like HCT for those with truly bad MR-related variants but not for the others—though it remains unknown whether HCT can reverse the biologic implications of poor-risk AML.

To put it in perspective, Jayastu et al. analyzed the prognostic impact of “splicing factor” (SF) variants like *SRSF2, U2AF1, SF3B1* and *ZRSR2* in 994 subjects with newly-diagnosed AML, 27% of whom had SF mutations [5]. This study reported worse outcomes in subjects with SF variants receiving intensive chemotherapy. However, in those receiving venetoclax-based therapies survival was comparable in those with and without SF mutations [Intensive chemotherapy+venetoclax: 19.6 *versus* 30.7 months, *p* = 0.98; low intensive chemotherapy+venetoclax: 12.3 *versus* 8.5 months, *p* = 0.14). Although this study did not parse the prognostic impact of individual MR-variants, it does suggest venetoclax may alter the prognosis of people with AML with SF variants and could represent an initial intervention.

In the ELN 2024 genetic risk classification of adults with AML receiving less-intensive therapies like venetoclax, the median survival of people with MR-variants but without signaling variants (*FLT3*-ITD negative*, KRAS/NRAS* wildtype) resembles that of people with favorable-risk variants, whereas those with MR-variants and co-occurring signaling variants had survivals like those with intermediate-risk mutations [6, 7]. MR-variants do not appear to correlate with adverse risk in those receiving venetoclax-based therapies, indicating the context of the crime is also important.

In conclusion, not all MR-variants carry equal prognostic weight in AML, and they are context-dependent including the size of the variant clone, therapy and the other members of the gang, and are deserving of a trial by their peers.

## References

[1] Foster MA. Mens Rea: An Overview of State-of-mind Requirements for Federal Criminal Offenses. Congressional Research Service. 2021.

[2] Sekeres MA, Guyatt G, Abel G, Alibhai S, Altman JK, Buckstein R, et al. American Society of Hematology 2020 guidelines for treating newly diagnosed acute myeloid leukemia in older adults. Blood Adv. 2020;4:3528–49. 32761235 10.1182/bloodadvances.2020001920 PMC7422124

[3] Döhner H, Wei AH, Appelbaum FR, Craddock C, DiNardo CD, Dombret H, et al. Diagnosis and management of AML in adults: 2022 recommendations from an international expert panel on behalf of the ELN. Blood. 2022;140:1345–77. 35797463 10.1182/blood.2022016867

[4] Bill M, Eckardt J-N, Dohner K, Röhnert M-A, Rausch C, Metzeler KH, et al. Differential prognostic impact of myelodysplasia-related gene mutations in a European cohort of 4978 intensively treated AML patients. Leukemia 2026;40:63–71. 10.1038/s41375-025-02781-6 PMC12789037 41145671

[5] Senapati J, Urrutia S, Loghavi S, Short NJ, Issa GC, Maiti A, et al. Venetoclax abrogates the prognostic impact of splicing factor gene mutations in newly diagnosed acute myeloid leukemia. Blood. 2023;142:1647–57. 37441846 10.1182/blood.2023020649

[6] Döhner H, Pratz KW, DiNardo CD, Wei AH, Jonas BA, Pullarkat VA, et al. Genetic risk stratification and outcomes among treatment-naive patients with AML treated with venetoclax and azacitidine. Blood. 2024;144:2211–22. 39133921 10.1182/blood.2024024944 PMC11600046

[7] Döhner H, DiNardo CD, Appelbaum FR, Craddock C, Dombret H, Ebert BL, et al. Genetic risk classification for adults with AML receiving less-intensive therapies: the 2024 ELN recommendations. Blood. 2024;144:2169–73. 39133932 10.1182/blood.2024025409

